# Circulating Glutathione Peroxidase-3 in Elderly—Association with Renal Function, Cardiovascular Mortality, and Impact of Selenium and Coenzyme Q_10_ Supplementation

**DOI:** 10.3390/antiox13121566

**Published:** 2024-12-19

**Authors:** Jan Alexander, Jan Olav Aaseth, Lutz Schomburg, Thilo Samson Chillon, Anders Larsson, Urban Alehagen

**Affiliations:** 1 Norwegian Institute of Public Health, N-0213 Oslo, Norway; 2 Research Department, Innlandet Hospital Trust, N-2381 Brumunddal, Norway; jaol-aas@online.no; 3 Institute for Experimental Endocrinology, Charité-Universitätsmedizin Berlin, D-10115 Berlin, Germany; lutz.schomburg@charite.de (L.S.); thilo.chillon@charite.de (T.S.C.); 4 Department of Medical Sciences, Uppsala University, SE-751 85 Uppsala, Sweden; anders.larsson@medsci.uu.se; 5 Division of Cardiovascular Medicine, Department of Health, Medicine and Caring Sciences, Linköping University, SE-581 85 Linköping, Sweden

**Keywords:** glutathione peroxidase-3, micronutrients, supplementation, elderly, renal function, cardiovascular mortality, selenium, coenzyme Q_10_

## Abstract

Low-selenium status was associated with impaired renal function, which improved after selenium and coenzyme Q_10_ supplementation in an RCT. Here, we evaluated serum glutathione peroxidase-3 (GPx3) and its relation to serum selenium, selenoprotein P (SELENOP), renal function, mortality, and the impact of supplementation, which are all important, especially in elderly individuals. In total, 383 study participants (197 receiving selenium yeast and coenzyme Q_10_ and 186 on a placebo) were evaluated. We applied benchmark dose modelling to determine GPx3 saturation, ANCOVA, Kaplan–Meier, and multivariate Cox proportional regression analyses for mortality evaluations. Selenium and GPx3 activity were modestly correlated. In comparison with SELENOP, GPx3 levelled off at a much lower value, 100 vs. 150 µg Se/L. GPx3 was associated with renal function, but not SELENOP. Supplementation increased glomerular function by ≈23% with an increase in GPx3. Being low in GPx3 displayed twice the risks of mortality in both placebos and active treatments. At serum selenium <100 µg/L, GPx3 activity was dependent on both selenium status and renal function. As renal function is reduced in the elderly, GPx3 is not an appropriate marker of selenium status. Low GPx3 was associated with an increased risk of mortality dependent of selenium status and independent of renal function.

## 1. Background

Selenium is an essential trace element that is incorporated as selenocysteine in selenoproteins. In humans, there are 25 genes coding for selenoproteins [1,2,3]. The high inherent nucleophilicity and reactivity with electrophiles of the selenide group serve as a basis for the important functions of selenoenzymes that include protection against oxidative stress, mediation of redox regulation, thyroid hormone deiodinase activity, and control of protein misfolding [3,4,5]. Selenium content in the soil varies greatly between different regions and hence also in locally produced food and dietary intake, which in some regions may result in selenium deficiency or low intakes [2,6]. Mainland Europe and Scandinavia are low-intake areas, while the USA is an area with higher intake [2,7]. There is a large variation among different regions of the world in the selenium content of locally cultivated dietary products. Consequently, habitual selenium intake varies, which may result in selenium deficiency or suboptimal intakes in many populations in areas with low-selenium soil, while inhabitants in areas with soil high in selenium may experience the toxic effects of selenium [2,8]. A tendency of increased use of locally produced food may further accentuate regional differences. For example, the estimated selenium intake in the Nordic and Baltic area varies from about 40 to 90 µg selenium/day. Higher intake levels are found in Finland, where fertilisers are systematically enriched with selenium [1].

In regions with low-selenium intake, inhabitants appear to have an impaired protection against oxidative stress and thyroid dysfunction [9,10,11,12,13,14], apparently being associated with a higher risk of inflammation and, i.a., cardiovascular and renal diseases [1,15,16,17,18,19].

Coenzyme Q_10_ (CoQ_10_) is both a component of an electron transport chain and a lipid-soluble antioxidant, which is reduced in old age [20,21]. Its antioxidant function, protecting lipid membranes and lipoproteins, is dependent on the supply of reducing equivalents from cytosolic selenoenzyme thioredoxin reductase 1 [22]. In a recent study, selenium, in a mechanism independent of selenoprotein synthesis and via hydrogen selenide, appeared to reduce CoQ_10_ in mitochondria, thereby protecting them against lipid peroxidation, dysfunction, and cell death by ferroptosis [23].

Glutathione peroxidase-3 (GPx3) is an actively secreted extracellular selenoprotein. It eliminates hydrogen peroxide and complex hydroperoxides, i.a., phosphatidyl choline peroxide but apparently not cholesterol-5α-hydroperoxide, in a reaction where glutathione (GSH), thioredoxin, or glutaredoxin is oxidised [24,25,26,27,28]. Its expression is upregulated by hypoxia and its encoding gene also contains antioxidant-, metal-, SP1-, PPAR-, and glucocorticoid-responsive elements [24,29]. Circulating GPx3 constitutes about 10–15% of serum selenium, second only to circulating SELENOP, which makes up about 50–60%, in selenium-replete humans [1,30]. SELENOP is secreted by the liver, with each molecule containing up to 10 selenocysteines, and transports selenium to peripheral tissues [3,31]; these include, i.a., the thyroid, brain, heart, kidney, and testes, of which the thyroid and kidney have the highest concentrations of selenium [32,33,34]. A surplus of selenium is detoxified and mainly excreted in urine as selenosugars and trimethyl selenonium ions [1].

Circulating GPx3 is mainly of renal origin. GPx3 is found in the renal cortex in the parietal cells of the Bowman’s capsule and the basement membrane of proximal tubuli from which it is secreted into plasma. GPx3, in lower amounts, can enter from plasma into the basement membranes of other tissues and is also found in the follicular space of the thyroid gland [35,36,37,38]. In plasma, it has been found in the HDL fraction [25]. GPx3 occurrence in serum is related to SELENOP as SELENOP and SELENOP fragments pass into primary urine and are subsequently reabsorbed in the proximal tubuli by low-density lipoprotein receptor-related protein 2 (LRP2 or megalin)-mediated endocytosis [1,30,39]. Reduced renal uptake of SELENOP is consequently reflected in suppressed GPx3 activity in blood, as observed in mice with impaired LRP2 expression [32]. Accordingly, the plasma’s GPx3 activity appears to be related both to selenium status and renal function as it is reduced in renal failure, and the reduction is associated with the severity [36,40]. In acute kidney failure following cardiac surgery, GPx3 is reduced and may serve as a prognostic factor for renal failure [41]. Results from studies in GPx3 knock-out and wild-type mice in a model of surgery-induced chronic kidney disease indicate that GPx3 deficiency contributes substantially to chronic kidney disease-induced cardiac failure/disease [42,43]. As even in a healthy ageing population renal function decreases with increasing age [44], the wide use of GPx3 as a biomarker for selenium status is not optimal in the elderly. An important mechanism appears to be depressed cardiac function because of coronary artery thrombosis, which accords with earlier studies on GPx3 knock-out mice displaying (increased bleeding time and) vascular platelet-dependent thrombosis and endothelial dysfunction [45]. GPx3 deficiency is associated with risk of stroke and embolism [46]. GPx3 has also been linked to cancer, diabetes, adipocyte differentiation, inflammation, and mortality [24].

As the concentrations of its cofactors GSH, thioredoxin, and glutaredoxin in plasma are very low, several orders of magnitude below those in cells, the physiological role of circulating GPx3 remains uncertain as it is less likely to have a catalytic antioxidant function in that milieu [24,27]. However, dependent on assay conditions, significant activity has been reported using physiological plasma concentrations of GSH [25], and one may also speculate that in the vicinity of endothelial cells where GSH is excreted, GSH might reach higher concentrations and support local GPx3 activity [36].

Both GPx3 and SELENOP have been used as indicators of selenium status [1,47,48]. GPx3 readily responds to selenium status and appears to reach a maximum level at a lower selenium concentration (about 70 to 90 µg selenium/L) than that of SELENOP, which is now considered a more appropriate marker of selenium status, as GPx3 in serum seems to be dependent also on renal function [1,49].

Previously, in a cohort of elderly Swedes low in selenium (median serum value 67 µg/L), we found that low-selenium status was associated with impaired renal function [19]. Furthermore, in this population, a randomised double-blind placebo-controlled prospective trial (RCT) was conducted showing that supplementation with selenium and coenzyme Q_10_ in comparison with a placebo caused an improvement in renal function determined as the estimated glomerular filtration rate (eGFR), using the Chronic Kidney Disease Epidemiology Collaboration (CKD-EPI) algorithm [19].

The aim of the present sub-study was to further characterise this elderly, community-living population low in selenium, with respect to serum GPx3 status and its relationship to serum selenium, SELENOP, and renal function. Furthermore, we wanted to investigate whether 4 years of supplementation with selenium and CoQ_10_ in comparison with a placebo would impact GPx3 status and its association with renal function and cardiovascular and total mortality after 10–12 years.

In addition, we intended to explore possible associations between GPx3 status and biomarkers of inflammation, oxidative stress, cardiac function, and thromboembolism.

## 2. Materials and Methods

### 2.1. Study Participants and Clinical Follow-Up

In 1998, all residents aged 69–88 in a rural municipality in Southeast Sweden (n = 1320) were invited to join a cardiovascular-focused epidemiological study, with 876 agreeing to participate. By 2003, of the surviving participants (n = 675), 443 community-dwelling individuals were recruited for a prospective randomised double-blind placebo-controlled trial. This study involved four years of supplementation with either selenium and coenzyme Q_10_ or a placebo, alongside biannual blood sample collections [50]. Participants received 200 mg/day of coenzyme Q_10_ capsules (Bio-Quinon 100 mg B.I.D, Pharma Nord, Vejle, Denmark) and 200 µg/day of organic selenium yeast tablets (SelenoPrecise 100 µg B.I.D, Pharma Nord, Vejle, Denmark) (n = 221), or a matched placebo (n = 222) over the course of 48 months, after which the intervention concluded. Participants continued any regular medications they were on, and compliance was measured by returning unused study medications. Each participant was assessed by one of three experienced cardiologists, who recorded clinical histories and conducted examinations both at the start and conclusion of this study. Measurements included blood pressure, New York Heart Association functional class (NYHA class), electrocardiograms (ECGs), and Doppler echocardiograms. Echocardiograms were performed with participants in the left lateral position, categorising ejection fraction (EF) readings into four classes with limits set at 30%, 40%, and 50% [51,52]. Normal systolic function was defined as EF ≥ 50%, while severely impaired function was EF < 30%. Only systolic function was assessed. Enrolment took place from January 2003 to February 2010.

**Figure 1:**
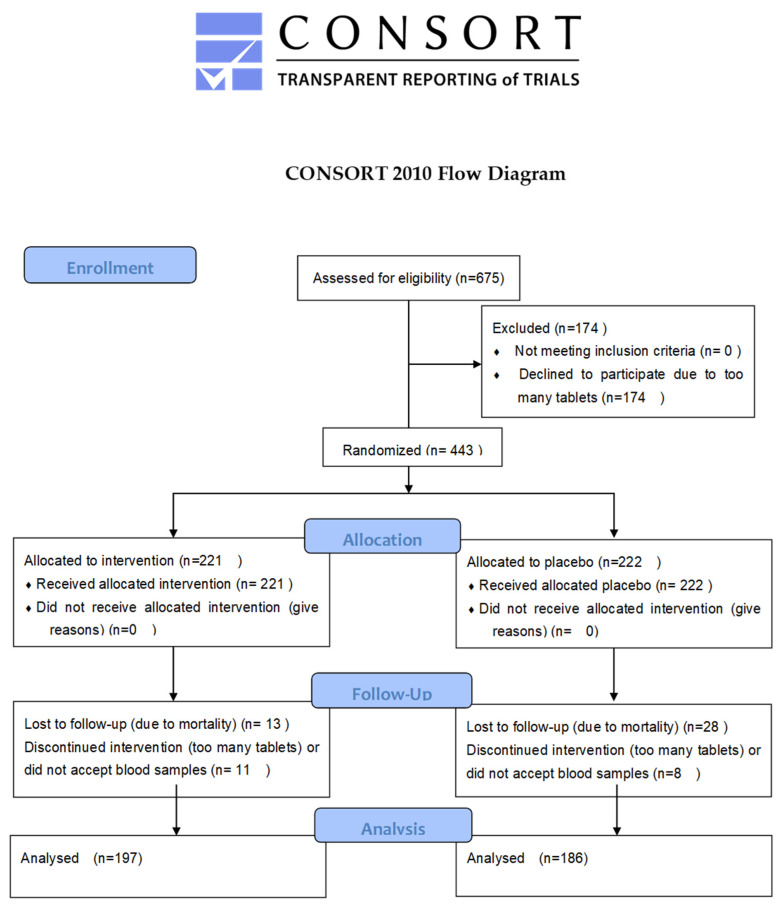
A flowchart of the total follow-up period.

For the sub-analysis of this study, only participants who provided blood samples throughout the entire intervention and survived the duration were included, resulting in a final population of 383 individuals: 197 received active supplementation with selenium and coenzyme Q_10_, while 186 were given a placebo (Table 1). A flowchart of the total follow-up period is presented as Figure 1.

### 2.2. Ethics Approval and Consent to Participate

This study was approved by the Regional Ethical Committee (Forskningsetikkommmitten, Hälsouniversitetet, SE-581 85 Linköping, Sweden; No. D03-176), and it conforms to the ethical guidelines of the 1975 Declaration of Helsinki. (The Medical Product Agency declined to review the study protocol since this study was not considered a trial of a medication for a certain disease, but rather one of food supplement commodities that are commercially available.) This study was registered at Clinicaltrials.gov, with identifier NCT01443780. Since it was not mandatory to register at the time this study started, this study has been registered retrospectively. Written, informed consent was obtained from all patients.

### 2.3. Blood Sampling

Blood samples were collected at inclusion in this study and, after 48 months, retrieved from participants resting in a supine position. Whole blood samples were collected in Vacutainer tubes containing 1/10 volume sodium citrate (0.11 mol/L). The vials for serum preparation were centrifuged at 3000× *g*, +4 °C. Serum and whole blood were frozen at −70 °C until analysis. No sample was thawed more than twice.

### 2.4. Determination of Selenium

The serum selenium analyses were performed using ICP-MS methodology on an Agilent 700 platform at Kompetenzzentrum für komplementärmedizinische Diagnostik, Zweigniederlassung der synlab MVZ Leinfelden GmbH (Leinfelden-Echterdingen, Germany). The accuracy of the measurements was checked by analysing two external reference materials with certified values of 63 μg/L and 103 μg/L (control programme offered by the Society for Advancement of Quality Assurance in Medical Laboratories, INSTAND e.V., Düsseldorf, Germany), showing values within 90–110% of certified concentrations. The precision of the method, checked by repetitive analyses of the same sera, showed an average coefficient of variation of 5.7%.

### 2.5. Determination of Selenoprotein P

Quantification of SELENOP concentrations in the serum samples was, as previously described [53], conducted using the selenOtest ELISA (selenOmed GmbH, Berlin, Germany), a chromogenic enzyme-linked immunosorbent assay (ELISA) specific to human SELENOP (lot STE.21002). Calibrators and controls were included in duplicates on each assay plate to standardise the method and monitor assay quality. Serum samples were diluted 1 to 40, processed according to the manufacturer’s instructions, and analysed by a 4-parameter logistic log function. The inter- and intra-assay CVs were below 5% during the analyses, as calculated from the control samples.

### 2.6. Determination of GPx3

Extracellular GPx3 activity was determined in the serum samples, as previously described [53], by a coupled enzymatic test monitoring NADPH consumption at 340 nm. Its decline is due to the coupled reaction, in which glutathione reductase regenerates glutathione that has been oxidised by GPx3 activity to reduce the added H_2_O_2_ substrate [54]. The reactions were started by adding H_2_O_2_ into the reaction buffer containing reduced glutathione, NADPH, and glutathione reductase along with serum samples of 5 µL each. NADPH decline was detected spectroscopically in the 96-well plates and converted to units of enzymatic activity as described. A serum control sample was included into each assay run for assessing the analytical quality, yielding inter- and intra-assay CVs of less than 15% during the analysis.

### 2.7. Renal Function

#### 2.7.1. Creatinine and Cystatin-C

Creatinine and cystatin-C were analysed on a Cobas c701 chemistry analyser (Roche Diagnostics, Rotkreutz, Switzerland) with reagents from the same manufacturer. The creatinine method used was enzymatic and calibrated by isotope dilution mass spectrometry. Cystatin-C was analysed with a particle-enhanced turbidimetric assay.

#### 2.7.2. Assessment of Renal Function

The estimated glomerular filtration rate (eGFR) was calculated, as previously described [19], based on creatinine and Cystatin C and combined using algorithms recommended by the Chronic Kidney Disease Epidemiology Collaboration (CKD-EPI), i.e., CKD-EPI-creat., CKD-EPI-CysC, and CKD-EPI-Comb.

### 2.8. Determination of Biomarkers

All blood samples were collected at the start of this study, and after 42 and 48 months, and they were drawn with the participants resting in a supine position. Pre-chilled EDTA vials for plasma were used. The vials were centrifuged at 3000× *g*, +4 °C, and they were then frozen at −70 °C. No sample was thawed more than twice.

### 2.9. NT-proBNP and Copeptin Analyses

NT-proBNP was measured using an electrochemiluminescence immunoassay (Elecsys 2010, Roche Diagnostics, Mannheim, Germany). The analytical range was 5–35,000 ng/L (0.6–4130 pmol/L). The total coefficient of variation was 4.8% at the level of 217 ng/L (26 pmol/L) (*n* = 70) and 2.1% at the level of 4261 ng/L (503 pmol/L) at our laboratory.

Plasma copeptin was measured on the Kryptor Compact platform (BRAHMS GmbH, Hennigsdorf, Germany). The inter-assay CVs are <15% at 20 pmol/L, <13% for 20–50 pmol/L, and <8% for concentrations >50 pmol/L according to the manufacturer; further assay validation has been reported previously [55].

### 2.10. Determination of MR-proADM

MR-proADM was analysed with the use of a commercially available assay on the Kryptor platform (BRAHMS). The inter-assay coefficient of variation was <20% for samples from 0.2 to 0.5 nmol/L, <11% for samples from 0.5 to 2 nmol/L, and <10% for samples from 2 to 6 nmol/L.

### 2.11. Determination of P-Selectin

Soluble P-selectin (sCD62P) was analysed utilizing an ELISA from R&D (Abingdon, UK). The intra-assay coefficient of variation (CV) was about 5% and the inter-assay CV about 9%.

### 2.12. Determination of ICAM-1 and Hepatocyte Growth Factor HGF

ICAM-1 (kit number DY720) and HGF (kit DY294) were analysed using commercially available sandwich enzyme-linked immunosorbent assay kits (ELISA) (R&D Systems, Minneapolis, MN, USA). The assays had a total coefficient of variation of approximately 6%.

### 2.13. Determination of D-Dimer

Whole blood samples were analysed utilising an automated micro-latex D-dimer reagent, MRX-143, from Medirox (Nyköping, Sweden) using ACL Top analyser (Instrumentation Laboratories, Milan, Italy). For a low control at mean concentrations of 0.39 mg/L (n = 917) and a high control at 0.96 mg/L (n = 526), the total imprecision was 7.3% and 2.9%, respectively.

### 2.14. Statistical Methods

Descriptive data are presented as percentages or mean ± SD. A Student’s unpaired two-sided *t*-test was used for continuous variables and the chi-square test was used for analysis of one discrete variable. Repeated measures of variance were used to obtain better information on the individual changes in the concentration of the biomarker analysed, compared to group mean values.

In the evaluation regarding a possible association between the activity of GPx3 and the serum level of selenium and biomarkers for inflammation, Pearson product–moment correlation analysis was performed. The impact of covariates besides active treatment on GPx3 activity at 48 months was investigated using analysis of covariance (ANCOVA) methodology. Kaplan–Meier and Cox proportional regression analyses were used to demonstrate CV and all-cause mortality during the follow-up period.

*p*-values < 0.05 were considered significant, based on a two-sided evaluation. All data were analysed using standard software (Statistica v. 13.2, Dell Inc., Tulsa, OK, USA).

The relationships between serum selenium concentrations and the activities of GPx3, respectively, were modelled and the serum level at which maximum activity GPx3 occurred was determined using the benchmark dose modelling online instrument BMDS, United States Environmental Protection Agency (2021). BMDS Online (Build 2BE68256DAEE; Model Library Version 2021.09), https://bmdsonline.epa.gov/, accessed 1 October 2021.

## 3. Results

### 3.1. Association Between GPx3, Selenium, and SELENOP in Serum and Effect of Selenium and Coenzyme Q_10_ Supplementation

At inclusion, before the intervention, in the whole population (n = 383), GPx3 in serum varied from 113 to 320 U/L with a mean value of 230 U/L and a median value of 231 UL. The 25th and 75th percentiles were 205 and 256 U/L, respectively.

At baseline, selenium and GPx3 in serum were only modestly correlated (r = 0.31, *p* < 0.001) in the whole population (n = 383). As the mean serum selenium (S-Se) value was below 70 µg/L and most values were <100 µg/L, only in the few individuals with the highest serum selenium values would GPx3 activities be expected to reach a maximum.

**Figure 2:**
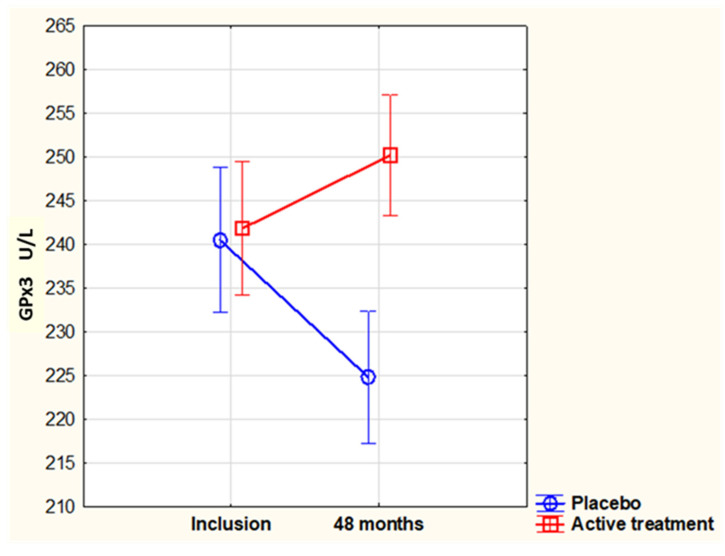
Activity of GPx3 in the study population at inclusion and after 48 months. The group receiving selenium and coenzyme Q_10_ is compared with the placebo group by repeated measures of variance methodology. Current effect: F(1, 147) = 18.3, *p* < 0.0001. Vertical bars denote 0.95 confidence intervals. Blue curve: placebo; red curve: active treatment group.

There was no difference in GPx3 at inclusion between the placebo and the active intervention group (227.2 vs. 232.0 U/L, *p* = 0.23). Neither did GPx3 change in the placebo group during 48 months of intervention (inclusion: 227.2 vs. 48 months: 224.2 U/L; *p* = 0.55). In the active intervention group receiving selenium and CoQ_10_, there was a significant increase in GPx3 (232.0 vs. 250.8 U/L, *p* < 0.0001), a value that was also significantly different from that of the placebo group at 48 months (250.8 vs. 224.2 U/L, *p* < 0.00001). By applying analysis by repeated measures of variation, a significant difference between inclusion and 48 months was obtained (Figure 1). In this analysis where only participants surviving for 48 months were included, the mean GPx3 values at inclusion were slightly higher than those for all participants included in each group (Figure 2).

**Figure 3:**
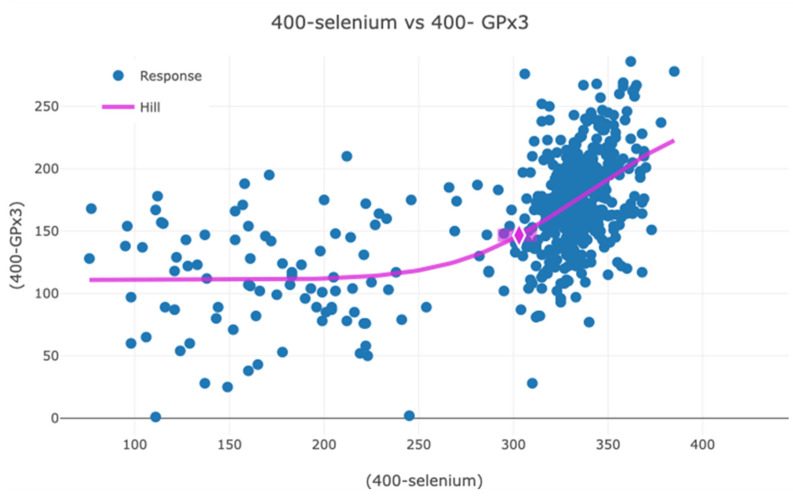
Benchmark modelling of the relationship between serum concentrations of selenium and GPx3 activity. Transformed data (selenium: 400 − serum selenium (µg/L); GPx3: 400 − serum GPx3 (U/L)) on the whole population measured at inclusion and at 48 months were used. Benchmark dose 10 (BMD10) for maximum expression of GPx3 is indicated (purple diamond). Modelling was conducted using US EPA BMDS package.

Following supplementation most of the participants in the active group had serum selenium concentration well above the concentration at which GPx3 is anticipated to reach a maximum (70–80 µg/L [1]), and there is no association between GPx3 and serum selenium. The serum selenium concentration at which the maximum activity of GPx3 occurred in this study was determined combining measurements made both at inclusion and after 48 months and using a benchmark modelling instrument for modelling the relationship between serum selenium and GPx3 [53]. The data were transformed as follows: serum selenium, 400 − serum selenium (µg/L); GPx3, 400 − GPx3 (U/L). The transformed data were then modelled, and a dose–response graph was obtained (Figure 3). Using a benchmark response (BMR) of 10%, a benchmark dose of 10% (BMD) for serum selenium of 100 µg/L (95% CI: 97–108 µg/L) was obtained for GPx3 saturation at a mean GPx3 concentration of 255 U/L. Even though saturation was reached, the GPx3 activity varied substantially, from 178 to 398 U/L.

Using GPx3 at 48 months in the total population as the dependent variable, we found in an ANCOVA that besides active supplementation with selenium and CoQ_10_, GPx3 activity at inclusion, and age were significant independent covariates. Renal function (CKD-EPI creat. incl.) was close to significant (Table 2).

**Figure 4:**
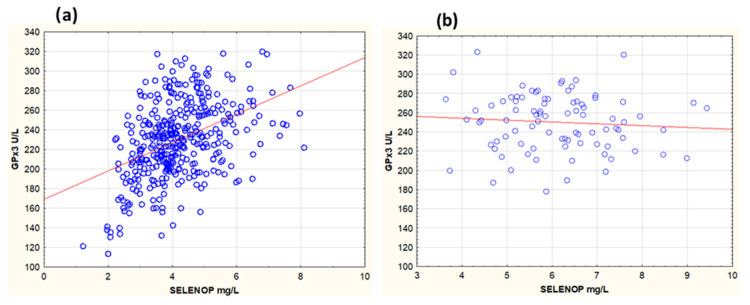
Serum activity of GPx3 in relation to serum concentration of SELENOP of the total population at inclusion (**a**) and the active treatment group (**b**) at 48 months. Notes: (**a**) r = 0.43, *p* < 0.001, r^2^ = 0.18; (**b**) r = −0.080, *p* = 0.46. Scales are different for x-axes in (**a**,**b**).

We also examined the relationship between GPx3 and SELENOP, the major circulating selenoprotein. At inclusion, in the whole population, both selenoproteins were correlated (r = 0.43, *p* < 0.001) (Figure 4a). At 48 months, in the placebo group there was no change in GPx3, which was still correlated with SELENOP (r = 0.54, *p* < 0.001). However, in the active treatment group when both GPx3 and SELENOP had reached saturation [53], GPx3 and SELENOP were not correlated (r = −0.08, *p* = 0.46) (Figure 4b).

### 3.2. Association Between GPx3 and Biomarkers of Oxidative Stress, Inflammation, Endothelial Function, and Thromboembolism 

We have previously reported results for biomarkers of oxidative stress and inflammation [9,16,56,57]. Upon investigation of the relationship between GPx3 and biomarkers of oxidative stress at inclusion, we found modest but significant inverse correlations in serum with MR-proADM (r= −0.36, *p* ≤ 0.001) and copeptin (r= −0.27, *p* < 0.001). Regarding markers of inflammation, GPx3 correlated modestly with HGF (r = 0.22, *p* = 0.31), while the correlations were modest and inverse for ICAM-1 (r = −0.35, *p* = 0.001) and P-selectin (r = −0.34, *p* = 0.001) and not significant for CRP. There was a weak inverse correlation between GPx3 and D-dimer (thromboembolism) (r = −0.11, *p* = 0.026), but no correlation with PAI-I (thromboembolism), and none with vWf (endothelial function).

### 3.3. Association with Biomarker of Myocardial Wall Tension

We observed weak but significant inverse correlations between GPx3 and NT-proBNP, which is a marker of myocardial wall tension, at inclusion in both the placebo (r = −0.20, *p* = 0.005) and the active treatment group (r = −0.22, *p* = 0.003). At 48 months, no correlations were observed.

### 3.4. Association Between GPx3 and Renal Function

We previously found that serum selenium was related to renal function [19]. Upon supplementation, there was a significant increase (≈25%) in the eGFR, and the fraction with reduced renal function was reduced by about 50% in the active treatment group. There were no significant changes in parameters of renal function in the placebo group [19] (Table 3).

Previous studies have observed that GPx3 is reduced in renal failure [58,59,60]. Hence, we examined whether GPx3 was related to serum creatinine and cystatin C in the whole population at inclusion and found that GPx3 was inversely correlated with both creatinine and more strongly with the cystatin C (creatinine: r = −0.22, *p* = 0.006; cystatin C: r = −0.35, *p* < 0.001).

As a parameter for renal glomerular function (estimated glomerular filtration rate, eGFR), we used the CKD-EPI equation based on creatinine and cystatin-C or both [19]. A stronger correlation with GPx3 was obtained when using cystatin-C than creatinine or both (CKD-EPI CysC: r = 0.35, *p* < 0.001; CKD-EPI Creat: r = 0.20, *p* = 0.015; CKD-EPI creat/CysC: r = 0.30, *p* < 0.001).

At 48 months, in the total population (n = 159) we observed that GPx3 correlated weakly with renal function (CKD-EPI CysC: 0.24, *p* = 0.002; CKD-EPI creat: r = 0.18, *p* = 0.023; CKD-EPI creat/CysC: r = 0.29, *p* < 0.001). Also, in the active group (n = 79) GPx3 was associated with renal function (CKD-EPI CysC: r = 0.26, *p* = 0.022; CKD-EPI creat: r = 0.26, *p* = 0.019; CKD-EPI creat/CysC: r = 0.27, *p* < 0.016), while no significant correlations were found between GPx3 and parameters of renal function in the placebo group. Renal function at inclusion did not impact GPx3 at 48 months.

We also examined whether the increase in GPx3 following supplementation was associated with the improvement in the eGFR and found that the increase in GPx3 from inclusion to 48 months was associated with a corresponding increase in CKD-EPI Cys C (r = 0.17, *p* = 0.033). Upon stratification into tertiles of GPx3 at inclusion, supplementation significantly increased the eGFR (CDK-EPI cys C) in all tertiles to a similar relative extent (21 to 26%) (tertile 1: *p* = 0.014; tertile 3: *p* = 0.051).

We further examined the relationship between serum SELENOP at inclusion, a measure of selenium status, and the same biomarkers of renal function (creatinine and cystatin C and eGFR) as above. However, SELENOP did not correlate with any of these.

### 3.5. Association Between GPx3 Activity at Inclusion and Mortality

As participants of both groups surviving for 48 months seemed to have higher mean GPx3 values at inclusion, we investigated the relation between having low GPx3 activity and mortality. Participants in the placebo and in the active treatment groups were stratified into quartiles according to GPx3 activity at inclusion and followed up for 12 years with respect to cardiovascular death. In the lowest quartile of GPx3 activity (Q1), 30 out of 48 participants (63.0%) in the placebo group suffered a CV death whereas the corresponding number in Q2–4 was 44 out of 103 (42.7%) (Chi^2^: 5.13, *p* = 0.024). Among the participants in the active treatment group in Q1, a total of 17 out of 47 participants (36.2%) suffered a CV death, whereas in Q2–4, the corresponding number was 28 out of 139 participants (20.1%) (Chi^2^: 4.92, *p* = 0.03).

**Figure 5:**
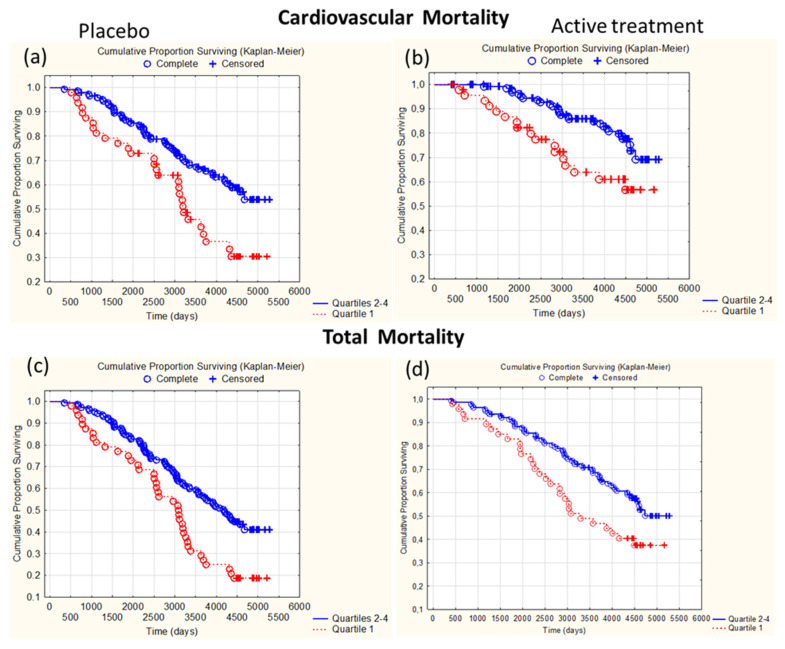
Kaplan–Meier graphs illustrating survival during 12 years from CV mortality (**a**,**b**) and total mortality (**c**,**d**) in the placebo group (**a**,**c**) and active treatment group (**b**,**d**), comparing those with GPx3 activity in the 1st quartile (Q1) with those in quartiles 2–4 (Q2–4) during a follow-up period of 12 years. Notes: (**a**) Z = 2.850267, *p* = 0.0044; (**b**) Z = 3.012643, *p* = 0.0026; (**c**) Z = 3.077865, *p* = 0.0021; (**d**) Z = 2.539015, *p* = 0.011.

Conducting a Kaplan–Meier analysis, a significantly higher CV mortality was observed in Q1 in comparison with Q2–4 of the placebo group (*p* = 0.0044) (Figure 5a) as well as in the active treatment group (*p* = 0.0026) (Figure 5b). However, in comparison with in the placebo group, a lower CV mortality in Q1 and Q2–4 was observed in the active treatment group (Figure 5a,b).

All-cause mortality was also examined using the same strata and a follow-up time of 12 years. In the lowest quartile (Q1), 39 out of 48 participants (81.3%) died, whereas in Q2–4, 63 out of 103 (61.2%) died (Chi^2^ 6.03, *p* = 0.014). Among the participants in the active treatment group in Q1, 29 out of 47 participants (61.7%) died whereas in Q2–4, 61 out of 139 (43.9%) died (Chi^2^: 4.46, *p* = 0.035).

In a Kaplan–Meier analysis, we found a significantly higher all-cause mortality in Q1 in comparison with Q2–4 in the placebo group (*p* = 0.0021), (Figure 5c) as well as in the active treatment group (*p* = 0.011) (Figure 5d). However, in comparison with the placebo group, a lower all-cause mortality in Q1 and Q2–4 was observed in the active treatment group (Figure 5a,b).

The risks of 12-year CV mortality associated with low GPx3 (<quartile 1), defined as <204 U/L in the placebo group and <205.9 U/L in the active treatment group, were addressed in Cox proportional hazard analyses using a multivariable model adjusting for several covariates impacting CV mortality (Table 4). In the placebo group, low GPx3 activity significantly increased the risk ratio of CV mortality (HR: 1.95, *p* = 0.008) besides diabetes (HR:1.69, *p* = 0.04). Remarkably, a similar increased risk was also observed in the active treatment group (HR: 2.07, *p* = 0.025). Notably, the renal function, measured as the eGFR, did not impact CV mortality in either group despite the renal origin of circulating GPx3. Notably, as mentioned, the main source of GPx3 is the proximal tubular cells.

## 4. Discussion

In the present study, we explore the relationship between serum GPx3, selenium, and SELENOP in addition to health outcomes like renal function as well as CV and all-cause mortality in an elderly community-living population low in selenium. The effect of supplementation with selenium and CoQ_10_ was investigated in an RCT. The main findings are the observations that GPx3 is positively associated with renal function and inversely with CV and all-cause mortality.

The median serum GPx3 activity of the present population was higher by approximately 90 U/L (63%) than the median value observed in the recently published Newcastle 85+ Study [61]. (GPX3 was determined in the same laboratory.) Also, serum selenium in that study was lower than in the present study at inclusion, with the median value being 53.6 vs. 67.0 µg/L. It is well known that dietary selenium is also generally lower in older people [61] and in Britain [7]. The population recruited in the Newcastle 85+ Study was about 8 to 9 years older than the mean age of the present population and deteriorating renal function in old age might also contribute to the lower GPx3 activity [62].

Our results accord well with the fact that circulating GPx3 is mainly of renal origin [37]. The GPx3 activity in serum is expected to depend both on the supply of selenium and the ability of the kidneys to synthesise GPx3 [1,30]. Selenium is mainly provided to the kidneys in the form of liver-derived SELENOP and SELENOP fragments found in primary urinary filtrate taken up via receptor-mediated endocytosis [1,30,32]. The current population had a low-selenium status, and it is therefore expected that serum GPx3 at inclusion is mainly dependent on selenium status as most of the participants have a normal or less severe reduced renal function (Table 1). Our observations showing an association between GPx3 and both selenium and SELENOP in serum at inclusion and at 48 months in the placebo group is therefore in line with the view expressed above. However, the association between GPx3 and various parameters of renal function indicates that renal function also influences serum GPx3 activity. The associations between GPx3 and parameters of renal function mainly reflecting glomerular filtration capacity and to a lesser extent tubular function were not very strong probably because GPx3 is mainly of tubular origin [37]. Notably, SELENOP at inclusion was not associated with any parameter of renal function.

Upon supplementation, resulting in a mean estimated intake of about 235 µg Se/day that is well above the level required to achieve saturation of GPx3 [1], serum GPx3 activity increased substantially and saturation of GPx3 was reached for participants of the active treatment group. We used benchmark modelling to determine the selenium concentration at which the maximum expression of GPx3 occurred. A value of 100 µg/L was obtained, which is slightly above the previously reported (70–90 µg/L) level [1]. This may partly be attributed to selenomethionine in the supplement being non-specifically incorporated into serum proteins [1]. The mean GPx3 activity was 255 U/L with a substantial variation.

As we found no association between GPx3 and SELENOP (Figure 4b) or selenium following active supplementation, the variation in GPx3 among supplemented participants could not be attributed to variation in selenium supply. Rather, it appears to depend on the ability of the kidneys to synthesise and secrete GPx3. In support of this we found, despite a relatively low number of surviving participants (n = 79), that GPx3 activity was associated with renal glomerular function. Likely, renal glomerular function to some extent is correlated with tubular function. However, in the placebo group no significant association between renal function and GPx3 was observed at 48 months, possibly due to the low number of participants (n = 74) and low-selenium intake still being the main determinant for GPx3 activity.

We have previously reported that supplementation with selenium and CoQ_10_ was associated with an improvement in kidney function [19]. Here, we found that in the active treatment group both renal function and GPx3 increased, and moreover, the increase in GPx3 activity was associated with a corresponding increase in renal function. This further strengthens the notion that GPx3 activity is causally related to renal function. This concurs with results from a study from Poland which reported that the reduction in plasma GPx activity was closely related to the severity of chronic renal failure patients when stratified into four categories from incipient, moderate, advanced, and end stage according to serum creatinine [49]. Their control group consisted of healthy individuals in the age range 23 to 64 years. Significant reduction in plasma GPx was even observed in patients with incipient failure. The elderly community-living population of our study was older than the control group of the Polish study, and the mean creatinine concentration of the present population corresponded to the mean creatinine concentration found in the group characterised as having incipient failure in the Polish study. This accords with the fact that renal function is reduced in old age [62]. Notably, it appears that the variation in normal and less severe reduced renal function of the present population is associated with variation in GPx3 activity. Hence, reduction in GPx3 is not only related to severely reduced renal function such as patients on haemodialysis [63,64,65] or patients with acute renal failure [41]. Of further interest is that supplementation in the present study improved renal function and also increased GPx3 in all three tertiles when stratifying according to GPx3, contrary to end-stage failure where supplementation, despite in some studies improving selenium status, did not raise or only weakly raised GPx3 [63,65,66].

NT-proBNP is a marker of myocardial wall tension that increases in heart failure and with age. It leads to increased diuresis and vascular dilatation [16] and is known to be inversely associated with renal function [67]. We have previously reported that NT-proBNP is related to selenium in the current population [50]. Here, we found that at inclusion, this biomarker was inversely associated with GPx3, which might indicate relations both to selenium status and renal function.

Studies in the literature have reported that selenium and/or GPx3 is inversely associated with oxidative stress [68,69,70], inflammation [24,71], endothelial function [42,72,73,74], and thromboembolism [24,42,45,46,75]. We found negative associations between GPx3 and MR-proADM and copeptin indicating a positive relation to reduced oxidative stress. Inverse correlations between GPx3 and ICAM-1 and P-selectin and positive correlation with HGF indicate a positive relation to reduced inflammation. While there was no relation to biomarkers of endothelial function, a weak inverse association with D-dimer was in accord with previous reports on a tendency of thromboembolism in GPx3 deficiency.

We further explored the relationship between GPx3 and CV and all-cause mortality during a follow-up time of 12 years. When stratifying into quartiles according to GPx3 activity and comparing participants in Q1 with those in Q2–4, those in Q1 had a significantly higher mortality. This was the case in both the placebo group and the active intervention group; however, in the latter the mortality was reduced in subjects both in Q1 and Q2–4. Similar findings were obtained when examining all-cause mortality (Figure 4). The increased CV mortality risk was further examined using a multivariate Cox proportional regression analysis in the placebo and active intervention group to quantify the relative risks associated with having low GPx3 at inclusion and the impact of other known risk factors, in particular renal function. Although the intervention resulted in a maximised GPx3 activity, improved renal function, and generally raised CV survival as observed in the Kaplan–Meier plots (Figure 4), the relative risks of having low GPx3 at inclusion were still similar in both groups. Only diabetes mellitus was a significant variable in the placebo group. Renal function had no impact in either group. We think this finding of having low GPx3 being associated with CV mortality independent of selenium status and renal function is novel and remarkable. Our findings concur with those from a nested case–control study from the Minnesota Heart Survey where it was found that GPx3 was inversely related to CV mortality, but this was confined to those having an HDL cholesterol value below the median [76]. As the physiological role of extracellular GPx3 is unclear [24], so are the underlying mechanisms of increased mortality risk related to low GPx3. It might be related, i.a., to impaired defence against extracellular oxidative stress and oxidation of LDL, as it has been reported that GPx3 was inversely correlated to the oxidised-LDL/HDL ratio in a cross-sectional study from Mexico [77]. Furthermore, low GPx3 has been related to CV disease (CVD) risk factors such as renal failure, increased inflammation, and increased tendency of platelet-dependent thrombo-embolism with an increased tendency of stroke [24,45,72].

Notably, there is a striking difference between being low in GPx3 and SELENOP, with regard to the association with CV and all-cause mortality in the same population [53]. While a low SELENOP at inclusion was associated with both an increased CV and all-cause mortality in the placebo group, this difference was wiped out in the supplemented group. This would indicate that low GPx3 at saturation is also a disease risk factor beyond being a marker of selenium status and renal function.

## 5. Limitations

The limited size population used in this study may increase the uncertainty of the results obtained. Evaluations were conducted in a two-step procedure, which was used in several of the analyses conducted to support the internal validity and reliability of the results obtained. As sample size was limited and the nature of this study is pioneering, the findings should be considered as hypothesis-generating.

A limitation is also that only one treatment group receiving selenium and CoQ_10_ combined, in addition to the placebo group, was used in this study due to limited resources when the original study was designed in 2002 and 2003. The rationale for combining selenium and CoQ_10_ was based on the study by Xia et al. in 2003 [22] showing that selenoenzyme thioredoxin reductase reduces and activates CoQ_10_ to ubiquinol, a pivotal lipophile antioxidant. The favourable choice of the supplement combination used received considerable support from a recent analysis indicating direct positive effects of Se on CoQ_10_ reduction and activity, which can proceed independent from selenoprotein biosynthesis and protect mitochondria from dysfunction and cells from death by ferroptosis [23]. A separate RCT comparing a selenium-only group with a CoQ_10_-only group would be needed for separating the effects of the two compounds.

As the study population represents both a narrow age stratum of elderly Caucasians living in Scandinavia and a population low in selenium, the results cannot be extrapolated to other age groups and populations with other selenium status and further different characteristics.

## 6. Conclusions

At serum selenium concentrations below 100 µg/L, the serum GPx3 activity was dependent on selenium status and renal function, while upon supplementation achieving maximum enzyme expression serum GPx3 was dependent on renal function and other regulating factors. The increase in GPx3 following supplementation was associated with improvement in renal function. SELENOP was not related to renal function nor to GPx3 at maximum expression. Low GPx3 was associated with an increased relative risk of CV and all-cause mortality independent of selenium status and renal function, with a similar relative risk in both groups; however, the risk was generally lower at a high-selenium status.

## Figures and Tables

**Table 1:** Baseline characteristics of the study population receiving active treatment or placebo during an intervention time of four years.

	Active	Placebo	*p*-Value
n	197	186	
Age Years, (SD)	76.9 (3.4)	77.1 (3.0)	
Males/Females n	100/97	101/85	
**History**			
Smokers (Present), n (%)	14 (7.1)	16 (8.6)	0.59
BMI, kg/m^2^ (SD)	26.8 (3.9)	27.1 (4.1)	0.68
Diabetes, n (%)	41 (20.8)	42 (20.7)	0.67
Hypertension, n (%)	140 (71.1)	143 (76.9)	0.20
IHD, n (%)	41 (20.8)	43 (23.1)	0.59
**Medications**			
ACEI, n (%)	33 (16.8)	41 (22.0)	0.19
ARB, n (%)	10 (5.0)	13 (6.4)	0.43
Betablockers, n (%)	69 (35.0)	62 (33.3)	0.73
Anticoagulants, n (%)	23 (11.5)	24 (12.7)	0.71
Diuretics, n (%)	61 (30.5)	74 (39.8)	0.71
Statins, n (%)	40 (20.3)	39 (21.0)	0.87
**Examinations**			
EF < 40%, n (%)	10 (5.1)	12 (6.5)	0.56
s-selenium, µg/L, (SD)	65.5 (15.9)	65.7 (18.0)	0.97
SELENOP, mg/L, (SD)	4.14 (1.10)	4.21 (1.22)	0.59
GPx3, U/L, (SD)	232.0 (37.7)	227.2 (39.2)	0.23
Creatinine, µmol/L (SD)	92.3 (26.8)	91.2 (30.6)	0.80
Cystatin C, mg/L (SD)	1.23 (0.31)	1.23 (0.34)	0.99
CKD-EPI, mL/min/1.73 m^2^ (SD)	61.4 (16.0)	64.7 (18.1)	0.16

Notes: ACEI: ACE inhibitor; ARB: Angiotensin receptor blocker; EF: ejection fraction; IHD: ischaemic heart disease; SD: standard deviation. Values are means ± SDs or frequency (percent). Student’s unpaired two-sided *t*-test was used for continuous variables and the chi-square test was used for analysis of one discrete variable.

**Table 2:** Analysis of covariance using GPx3 after 48 months as dependent variable.

Effect	Sum of Squares	Degrees of Freedom	Mean Squares	F	*p*
Intercept	695	1	695	0.87	0.35
GPx3 incl.	22,124	1	22,124	27.56	<0.0001
CKD-EPI creat. incl.	2875	1	2875	3.58	0.06
Age	4011	1	4011	5.00	0.03
Selenium incl.	703	1	703	0.88	0.35
Smoking	998	1	998	1.24	0.27
Active treatment	21,220	1	21,220	26.43	<0.0001
Corr. Hypertension	29	1	29	0.04	0.85
Corr. Diabetes mellitus	5	1	5	0.01	0.93
IHD	180	1	180	0.22	0.64
EF < 40%, incl.	516	1	516	0.64	0.42
Hb < 120 g/L	27	1	27	0.03	0.85
Error	97,945	122	802		

Note: CKD-EPI creat.: eGFR based on creatinine using algorithms of the Chronic Kidney Disease Epidemiology Collaboration; EF: ejection fraction; IHD: ischaemic heart disease.

**Table 3:** Renal function.

Group	Variable	Inclusion	48 Months	*p*-Value
**Active treatment**				
	CKD-EPI, mL/min/1.73 m^2^ (SD)	60.7 (16.4)	75.9 (21.4)	<0.0001
	CKD-EPI Cys C, mL/min/1.73 m^2^ (SD)	57.4 (16.4)	72.6 (21.1)	<0.0001
	eGFR < 60 mL/min/1.73 m^2^ (%)	46/91 (50.5)	20/91 (22.0)	0.0001
**Placebo**				
	CKD-EPI, mL/min/1.73 m^2^ (SD)	63.6 (17.6)	65.4 (15.9)	0.50
	CKD-EPI Cys C, mL/min/1.73 m^2^ (SD)	60.9 (18.0)	65.4 (20.5)	0.13
	eGFR < 60 mL/min/1.73 m^2^ (%)	36/82 (43.9)	28/82 (34.1)	0.20

The table shows renal function at inclusion and at 48 months of surviving participants in the active treatment group, n = 91, and the placebo group, n = 82, as reported before [19].

**Table 4:** Multivariate Cox proportional hazard regression analysis of relative cardiovascular mortality risk after 12 years of follow up of being in the lower quartile of GPx3 at inclusion.

**(a) Placebo Group**					
**Variable**	**β**	**β** **95% CI**	**HR**	**HR 95% CI**	***p*** **-Value**
GPx3 incl. Q1 < 204	0.67	0.17–1.16	1.95	1.19–3.19	0.008
Hyperlipidaemia	0.19	−0.32–0.69	1.21	0.73–1.99	0.47
Hb < 120 g/L	0.22	−0.36–0.80	1.25	0.70–2.23	0.45
Diabetes mellitus	0.52	0.022–1.02	1.69	1.02–2.78	0.04
Hypertension	0.07	−0.42–0.56	1.07	0.66–1.75	0.78
hsCRP incl.	0.004	−0.010–0.017	1.00	0.99–1.02	0.61
CKD-EPI Creat. incl.	0.005	−0.013–0.023	1.01	0.99–1.02	0.59
BMI	0.007	−0.039–0.054	1.01	0.96–1.06	0.74
**(b) Active Treatment Group**					
**Variable**	**β**	**β** **95% CI**	**HR**	**HR 95% CI**	***p*** **-Value**
GPx3 incl. Q1 < 205.9 U/L	0.73	0.090–1.37	2.07	1.09–3.93	0.025
Hyperlipidaemia	0.10	−0.62–0.81	1.10	0.54–2.25	0.79
Hb < 120 g/L	0.18	−1.15–0.79	0.83	0.31–2.19	0.71
Diabetes	0.34	−0.34–1.02	1.41	0.71–2.78	0.32
Hypertension	0.44	−0.27–1.14	1.55	0.76–3.14	0.23
hsCRP incl.	0.011	−0.013–0.034	1.01	0.99–1.03	0.37
CKD-EPI Creat. incl.	0.009	−0.033–0.015	0.99	0.97–1.01	0.46
BMI	−0.06	−0.14–0.025	0.94	0.87–1.03	0.17

Notes: Q1: 1st quartile, Hb: haemoglobin, HR: hazard ratio, CI: confidence interval CKD-EPI Creat.: eGFR calculated according to algorithm of Chronic Kidney Disease Epidemiology Collaboration, BMI: body mass index.

## Data Availability

Under Swedish Law, the authors cannot share the data used in this study and cannot conduct any further research other than what is specified in the ethical permissions application. For inquiries about the data, researchers should first contact the owner of the database, the University of Linköping. Please contact the corresponding author with requests for and assistance with data. If the university approves the request, researchers can apply to the Regional Ethical Review Board for the specific research question that the researcher wants to examine.

## Abbreviations

ACEI: ACE inhibitor; ANCOVA: analysis of covariance; ARB: Angiotensin receptor blockers, CKD-EPI: Chronic Kidney Disease Epidemiology Collaboration, CV: cardiovascular, CV: coefficient of variation, CVD: cardiovascular disease, EF: ejection fraction, ECG: electrocardiogram, eGFR: estimated glomerular filtration rate, GPx3: glutathione peroxidase-3, HR: hazard ratio, IHD: ischaemic heart disease, NYHA class: New York Heart Association functional class, SD: standard deviation, SELENOP: selenoprotein P.
